# Elevation of Interleukin-18 Correlates With Cardiovascular, Cerebrovascular, and Peripheral Vascular Events: A Cohort Study of Hemodialysis Patients

**DOI:** 10.1097/MD.0000000000001836

**Published:** 2015-10-23

**Authors:** Chih-Hsiang Chang, Pei-Chun Fan, Chan-Yu Lin, Chia-Hung Yang, Yi-Ting Chen, Su-Wei Chang, Huang-Yu Yang, Chang-Chyi Jenq, Cheng-Chieh Hung, Chih-Wei Yang, Yung-Chang Chen

**Affiliations:** From the Department of Nephrology, Kidney Research Center (C-HC, P-CF, C-YL, H-YY, C-CJ, C-CH, C-WY, Y-CC); Department of Cardiology, Chang Gung Memorial Hospital, Taipei (C-HY); College of Medicine (C-HC, P-CF, C-YL, H-YY, C-CJ, C-CH, C-WY, Y-CC, S-WC); Department of Biomedical Sciences (Y-TC); Clinical Informatics and Medical Statistics Research Center (CIMS), Chang Gung University, Taoyuan, Taiwan (S-WC).

## Abstract

Cardiocerebral vascular events are the major cause of mortality among patients with end-stage renal disease (ESRD). Subclinical inflammation and atherosclerosis have been implicated in the pathophysiology of ESRD. Evidence has shown the crucial role of interleukin-18 (IL-18) in inflammation. Interleukin-18 has been markedly upregulated in ESRD patients. Nevertheless, the ability of the IL-18 level to predict cardiocerebral vascular events and the correlation between IL-18 levels and cardiocerebral vascular events have not been established in hemodialysis patients.

To determine whether the serum IL-18 level predicts cardiocerebral vascular events, the authors studied 171 ESRD patients. Samples were collected and patients were followed for 24 months. Demographic data, the duration of hemodialysis, nutrition status, inflammatory parameters, dialysis adequacy, and lipid profiles were analyzed to predict the outcome by using multivariate logistic regression. Cutoff points were calculated by acquiring the highest Youden index. The Kaplan–Meier method was used to scrutinize the cumulative proportion of events.

The multivariate logistic regression model revealed that serum creatinine, C-reactive protein, and IL-18 levels were independent predictors for cardiocerebral vascular events. The odds ratio of events for each increase in IL-18 (pg/mL) was 1.008 for cardiocerebral vascular events. The area under the receiver operating characteristic curve of IL-18 was 0.779 ± 0.039, the overall correctness was 73%, and the Youden index was highest at a cutoff of 463 pg/mL. In the Kaplan–Meier model, patients with an IL-18 level higher than 463 pg/mL exhibited the highest probability of experiencing an adverse event during the entire follow-up period.

Increased serum IL-18 could be considered as a predictor of cardiocerebral vascular events in dialysis patients. It is noteworthy that various comorbidities might interfere the expression of IL-18; therefore, further validation study is required to incorporate IL-18 in clinical use.

## INTRODUCTION

Despite the improvement of medical technology, morbidity and mortality remain high in patients with end-stage renal disease (ESRD). Cardiovascular events are the major cause of mortality in dialysis patients, and atherosclerosis is one of the main reasons for cardiovascular events. Consistent evidence has shown that C-reactive protein (CRP) and proinflammatory cytokines, such as interleukin (IL)-1β, IL-6, and tumor necrosis factor-α are risk factors for atherosclerotic complications and predict death and adverse cardiovascular outcomes in dialysis patients.^1–4^ Interleukin-18 levels were shown to be positively correlated with CRP, which is a marker of acute-phase response that was originally identified as interferon-γ (IFN-γ), an inducing factor in Kupffer cells of the liver that plays a central role in the inflammatory cascade.^5,6^ Interleukin-18 has been demonstrated to be widely correlated with various diseases, such as sepsis, viral infection, systemic erythematous lupus (SLE), hepatitis, and malignancies.^7–16^ In acute kidney injury (AKI), IL-18 has been proposed to be a marker for early detection and outcome prediction in patients with acute myocardial infraction, heart failure, contrast nephropathy, cardiac surgery, and sepsis.^17–21^ In long-term dialysis patients, several investigations have shown that IL-18 is correlated with hospitalization, vascular injury, and all-cause mortality.^22–25^ The relationship between cardiocerebral vascular events and IL-18, however, has not been clearly identified. The objective of our study was to evaluate whether the serum IL-18 level is a useful indicator for assessing the risk of cardiocerebral vascular events in patients with ESRD receiving dialysis.

## MATERIALS AND METHODS

### Patient Information and Data Collection

This study was approved by the Institutional Review Board. Patients in this study received regular hemodialysis and clinical inspection in an out-patient hemodialysis center of tertially referral hospital in Taiwan from May 2009 to April 2011. All patients were enrolled in this study signed informed consent. Samples were collected during monthly biochemistry examinations in May 2009. Patients with the following conditions were excluded: lost follow-up during the 2-year study period; received regular dialysis for fewer than 3 months; had a history of organ transplant, or a recent infection (eg, any infectious condition recorded within 1 week before and after sampling). Finally, 171 patients were enrolled in the investigation. In this cohort, hemodialysis was provided 4 hours, 3 times per week. Dialyzers were single-use high-flux polysulfone-based membranes. Dialysates had a standard ionic composition with a bicarbonate-based buffer. Patient characteristics, including age, sex, hemogram data, biologic data, and the cardiothoracic ratio are listed in Table 1. The adequacy of dialysis was expressed as the weekly Kt/V_urea_ according to Daugirdas.^26^ Nutrition status was represented by the normalized protein catabolic rate (nPCR). All treatment for these patients were according to clinical practice guidelines through the National Kidney Foundation's Kidney Disease Outcomes Quality Initiative. A cardiovascular event was defined as a coronary arterial disease with angina, myocardial infarction, heart failure, or ventricular tachycardia/fibrillation documented in an Emergent room (ER) or upon admission by thallium scan or coronary angiogram. A cerebrovascular event was defined as a transient ischemic attack or ischemic or hemorrhagic stroke reported in an ER or upon admission. Transient ischemic attack was diagnosed according to definition by World Health Organization with further evidence provided by transcranial Doppler sonography/color carotid duplex.^27^ A vessel event was defined as a peripheral arterial disease and ischemic bowel identified using ultrasonography or angiography. The maximal event number for each patient was 1.

**TABLE 1:**
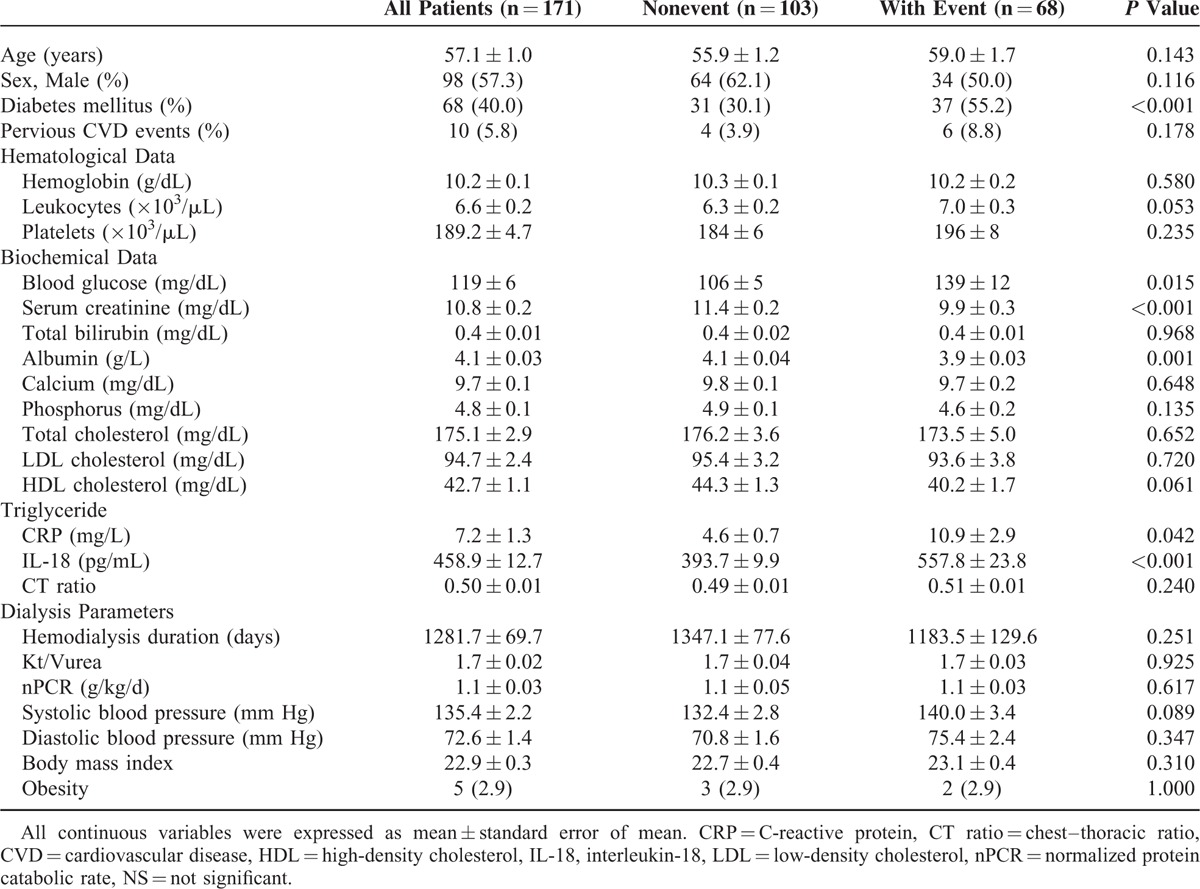
Demographic Data and Clinical Characteristics on the Sampling Day of According to With/Without Event in 2-Year Period Follow-Up

### Biochemical Parameters and Interleukin-18

Nonfasting blood samples were drawn from the arterial end of the vascular access immediately before hemodialysis was initiated. The samples were collected in vacutainer tubes containing ethylenediaminetetraacetic acid and then centrifuged at 2000 *g* for 5 minutes at 4 °C. Of the samples, 500 μL were separated for routine biochemistry by using standard laboratory techniques and an automatic analyzer, and the remainder was frozen at −80 °C until assayed.

Serum IL-18 was measured in duplicate by using a commercially available enzyme-linked immunosorbent assay (Category 7620, Medical and Biologic Laboratories, Nagoya, Japan) according to the manufacturer's instructions. If a variation between 2 single measurements was over 5%, then a third analysis was conducted.

### Statistical Analysis

The continuous variables were shown as mean and standard error. Kolmogorov–Smirnov test was used to analyze the normal distribution of the continuous variables. Student *t* test was used to compare the means of continuous variables with normal distribution. Categorical data were tested using the χ^2^ test. Correlation coefficients were calculated using Spearman rank analysis. Risk factors for 2-year events were assessed using univariate analysis. Age, sex, and variables that were statistically significant in the univariate analysis were included in the multivariate analysis by applying a multiple logistic regression based on backward elimination to obtain independent predictors. Hosmer–Lemeshow goodness-of-fit test was used for calibration. The discrimination was assessed analyzed using the area under the receiver operating characteristic curve (AUROC), which was compared using a nonparametric approach.^28,29^ The cutoff points were calculated by acquiring the highest Youden index.^30^ The benefit of reclassifying event risk was evaluated by comparing the predicted risk estimates between CRP (the base model) and IL-18 (the enhanced model). Events that were classified as nonevents in the base model and as events in the enhanced model represented a positive net reclassification improvement. In contrast, events that were classified as event in the base model and as nonevents in the enhanced model indicated a negative net reclassification improvement. For reclassifying the nonevents, the definition of net reclassification improvement was reversed. Finally, we calculated the Net reclassification improvement by summing up the net reclassification improvements for both the events and nonevents.^31^ Cumulative survival curves were assessed using a Kaplan–Meier approach. All statistical tests were 2-tailed and a *P* value <0.05 was considered statistically significant.

## RESULTS

### Characteristics of the Study Population

In total, 171 adult patients (98 men and 73 women) who received dialysis for a mean duration of 1281.7 ± 69.7 days were investigated. Patient characteristics, including age, sex, biologic, hematological, and dialysis parameter data are listed in Table 1. The mean patient age was 57.1 ± 1.0 years, and 42% of the patients were diagnosed with diabetes mellitus (DM). The mean IL-18 level was 458.9 ± 12.7 pg/mL, and the values were 393.7 ± 9.9 pg/mL and 557.8 ± 23.8 pg/mL with and without events, respectively. Table 1 provides patient characteristics according to events. No differences in sex, hemogram data, liver function, calcium, phosphorus, lipid profiles; and the CT ratio were evident. In addition, no differences in the hemodialysis duration and normalized protein catabolic rate were observed between the 2 groups. The event group contained significantly more patients with DM, high blood glucose, and CRP levels than did the control group. In addition, the patients in the event group exhibited lower serum creatinine (Cr) and albumin levels.

A multiple logistic regression model was applied to the overall study population to adjust for event-free periods according to age; sex; DM; blood glucose, Cr, albumin, CRP, and IL-18 levels. Only Cr, CRP, and IL-18 levels were independently associated with hospital admission according to the multivariate analysis (Table 2). The odds ratio for the first event for each pigogram per milliliter increase in the IL-18 concentrations was 1.008 [95% confidence interval (CI), 1.005–1.011; *P* < 0.001]. In another adjusted model, we adjusted for age, platelets, total cholesterol, hemodialysis duration, and systolic blood pressure, the result revealed that the independent effect of IL-18 remained (Appendix 1, http://links.lww.com/MD/A469).

**TABLE 2:**
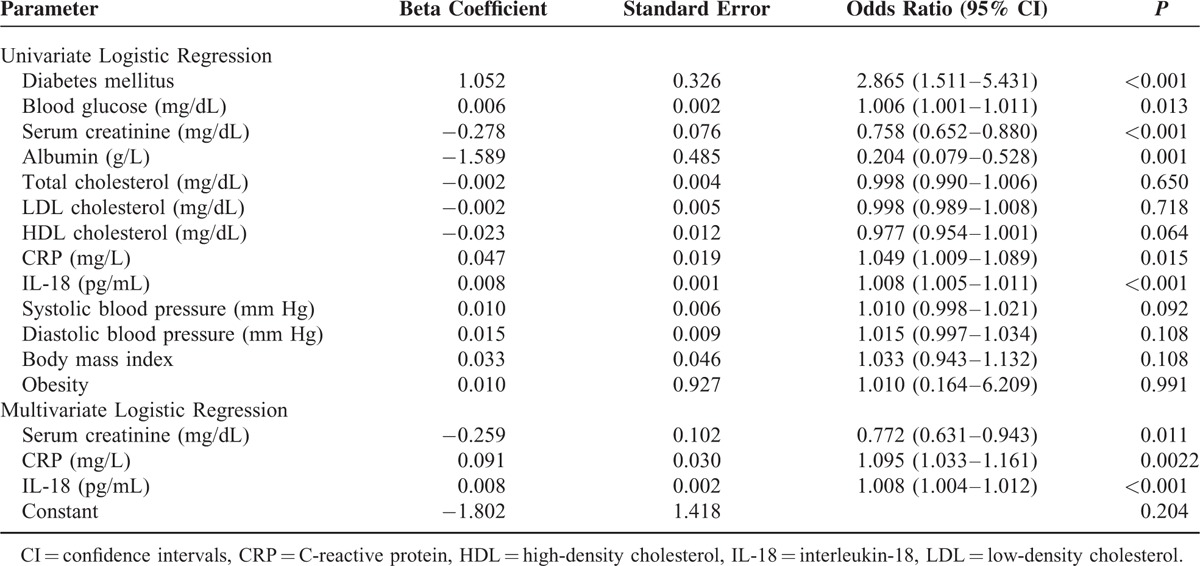
Logistic Regression Analysis for Event According to Baseline Prognostic Factors on the Sampling Day

### Interleukin-18 Concentrations and Future Cardiocerebral Vascular Events

**FIGURE 1:**
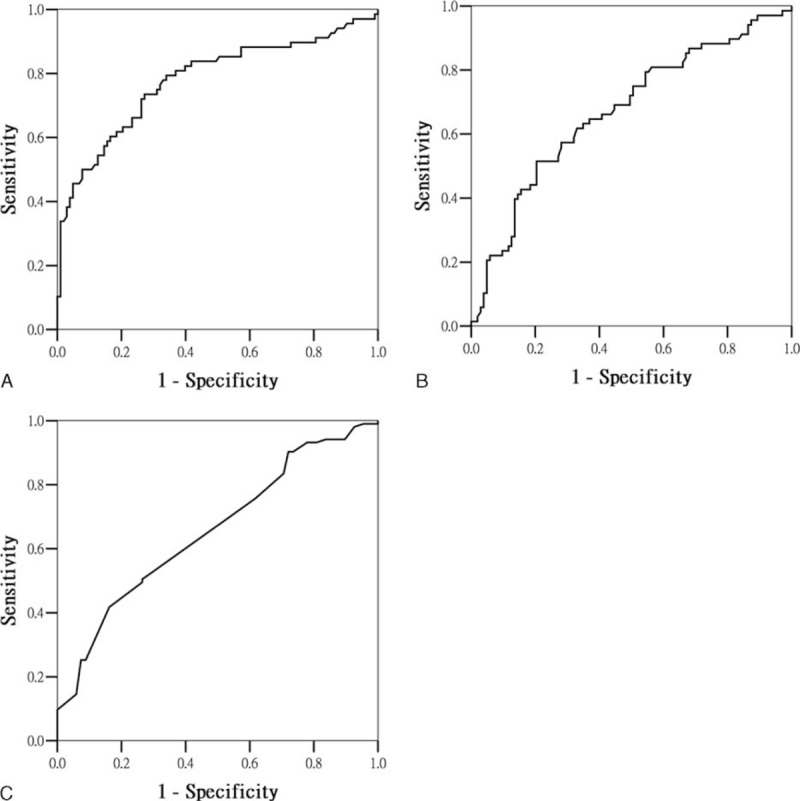
Area under operating curve according to different predictors. A, Interleukin-18 (AUROC 0.779 ± 0.39, 95% CI 0.703–0.855). B, C-reactive protein (AUROC 0.670 ± 0.043, 95% CI 0.568–0.754). C, Albumin (AUROC 0.656 ± 0.042, 95% CI 0.574–0.738). AUROC = area under the receiver operating characteristic curve; CI = confidence interval.

Median plasma concentrations of IL-18 at the baseline were significantly higher in patients who had experienced events than in those who had not (557.8 versus 393.7 pg/mL; *P* < 0.001). We compared the IL-18 level with CRP and albumin levels as a predictor of cardiocerebral vascular events in these patients. Figure 1 shows that the IL-18 level exhibited superior predictive ability (AUROC, 0.779 ± 0.039; 95% CI, 0.703–0.855), followed by CRP and albumin levels (AUROC, 0.670 ± 0.043; 95% CI, 0.568–0.754; AUROC, 0.656 ± 0.042; 95% CI, 0.574–0.738). To assess the predictive value of selected cutoffs for predicting 6-month mortality, the sensitivity, specificity, and overall correctness of prediction were determined. Interleukin-18 predicts further events with positive predictive value of 60% and negative predictive value of 83%. Table 3 summarizes the data calculated using the cutoff point providing the highest Youden index. The seru1m IL-18 level with a cutoff of 463 pg/mL exhibited the optimal Youden index and the highest overall correctness of prediction, and the albumin level exhibited the highest sensitivity when it was lower than 3.8 g/L. The positive net reclassification improvement was 0.295, and the negative net reclassification improvement was 0.165. The sum of clinical net classification was 0.13 compared with CRP (Appendix 2, http://links.lww.com/MD/A469).

**TABLE 3:**
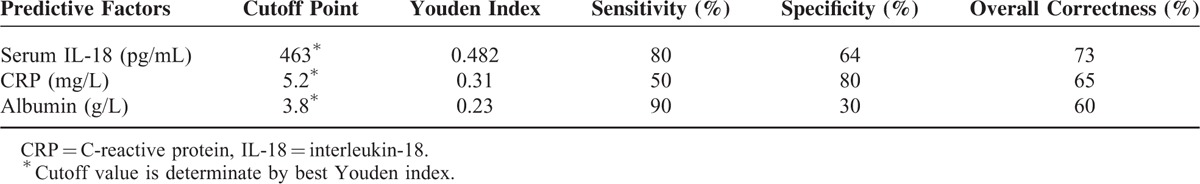
Predictive Ability of Event in Factor in End-Stage Renal Disease Patients

## DISCUSSION

Interleukin-18 is a 18-kDa molecule that was formerly called IFN-γ inducing factor, originally identified in endotoxin-challenged mice and cloned from activated macrophage and Kupffer cells as a proinflammatory cytokine.^32^ Caspase 1 converts the 24-kDa precursor into IL-18, which is an active monomer.^33^ Mice lacking caspase 1 and IFN-γ or mice neutralized by an IL-18 antibody are resistant to endotoxemia.^34^ Although IL-18 is biologically and structurally related to IL-1β, IL-18 appears to have unique features, especially in regulating the T helper type 1 (Th1) cell response via IFN-γ. Interferon-γ is a crucial producer of nitric oxide and provides a host for killing intracellular organisms. Interferon-γ has been used in adjuvant antibiotic therapy for mycobacterium infection. Because IFN-γ is essential for Th1, reducing IL-18 activity eliminates the Th1 response. Interleukin-18 binding protein is a natural inhibitor of IL-18, which is involved in immunosuppression in chronic kidney disease through the reduction of excretion.^35^

In human studies, elevated IL-18 levels have been observed to correlate with mortality and mobility in patients with AKI, infections, SLE, hepatitis, and malignancies. For AKI treated in coronary care units, serum and urine IL-18 levels exhibited discriminatory power in AKI and 6-month mortality.^17^ In patients with sepsis, the IL-18 level was associated with an unfavorable outcome and could be used to discriminate between gram-positive or gram-negative bacteremia.^14^ In the IL-18 gene located on chromosome 11 at 11q22.2-q22.3, which is close to linkage region for SLE in European populations, elevation of IL-18 levels was associated with gene polymorphism and disease activity.^7^ A high IL-18 level because of IL-18 gene (−137), G/C in a heterozygous condition led to a substantially increased risk of bladder cancer.^15^ In a previous study, the IL-18 level was shown to be negatively correlated with Kt/Vurea, meaning that levels of this cytokine are related to dialysis efficacy.^25^

Cardiocerebral vascular disease is a well-established cause of mortality in ESRD patients.^36,37^ Inflammation plays a crucial role in the pathophysiology of vascular damage. In ESRD, proinflammation mediators, such as IL-1β, IL-6, and tumor necrosis factor-α are implicated in both atheroma in inner layers and calcification in medium layers, which result in vascular damage, heart attack, or stroke.^38,39^ An increasing amount of evidence supports the immune reaction in ESRD, and many circulating cytokines involved in inflammation have been shown to predict morbidity and mortality in dialysis patients.^40^ Previous studies have shown that the IL-18 level correlates with hospitalization, vascular injury, and all-cause mortality.^22,24,25^ In patients receiving long-term dialysis, the relationship between cardiovascular events and IL-18 has not been clearly identified. Because IL-18 is a prominent inflammatory marker, researching its role in morbidity and mortality is vital. In this study, we demonstrated that elevated serum IL-18 levels predicted cardiocerebral vascular events in hemodialysis patients. The predictive power of the IL-18 level persisted even after adjustment for the effects of blood glucose, muscle mass (serum Cr), comorbidity (DM), the CRP level, and nutritional status (albumin). To the best of our knowledge, this is the first investigation that showed that the IL-18 level is a crucial predictor of cardiocerebral vascular events in the ESRD population.

Several mechanisms may explain the relationship between IL-18 levels and cardiocerebral vascular events in ESRD patients. First, studies have shown that IL-18 mediates atherosclerosis and cardiovascular death in patients exhibiting coronary artery disease.^41,42^ The IL-18 level is a major independent inflammatory predictor of 30-day major adverse cardiac events and unfavorable outcomes after acute myocardial infraction.^43^ In addition, the IL-18 level is associated with intracranial large artery stenosis, cerebral microbleeds, and recurrent stroke.^44–46^ Although a correlation between the IL-18 level and the outcome of peripheral arterial disease has not been proven, atherosclerotic and inflammatory processes remain the major cause of stenosis and thrombus formation.^47^ In addition, serum IL-18 levels were higher in ESRD patients than in a healthy population and were positively correlated with the serum level of homocysteine.^48^ Second, IL-18 contributes to vascular damage in ESRD patients.^24,49^ Finally, dialysis process-related microinflammation contributes to the production of IL-18 in ESRD. In our research, we identified a significant correlation between the CRP and IL-18 levels in our research population. These data were consistent with subclinical inflammation, which is usually observed in patients with ESRD.

Malnutrition, inflammation, and atherosclerotic (MIA) cardiovascular disease have been linked to high morbidity and mortality rates in hemodialysis patients.^50^ Our study illustrated that serum albumin and Cr levels were strongly prospective predictors (Table 2). To explore this relationship, we compared serum albumin and Cr values between patients who experienced events and event-free patients during the study period. We discovered that the mean albumin and Cr levels were significantly lower in the event group than in the nonevent group. These finding consisted with prior researches that explored the relationship between mortality, dialysis dosage, and Cr.^51^ Serum Cr reflects the status of lean body mass and Cr generation, and to a lesser extent, recent dietary protein intake. Chronic low levels suggest both decreased lean mass and probably decreased protein intake. Therefore, Cr represents a strong surrogate of health status instead of residual renal function. This reverse epidemiology was also seen in blood pressure, lipid profiles, and body mass index.^52,53^

**FIGURE 2:**
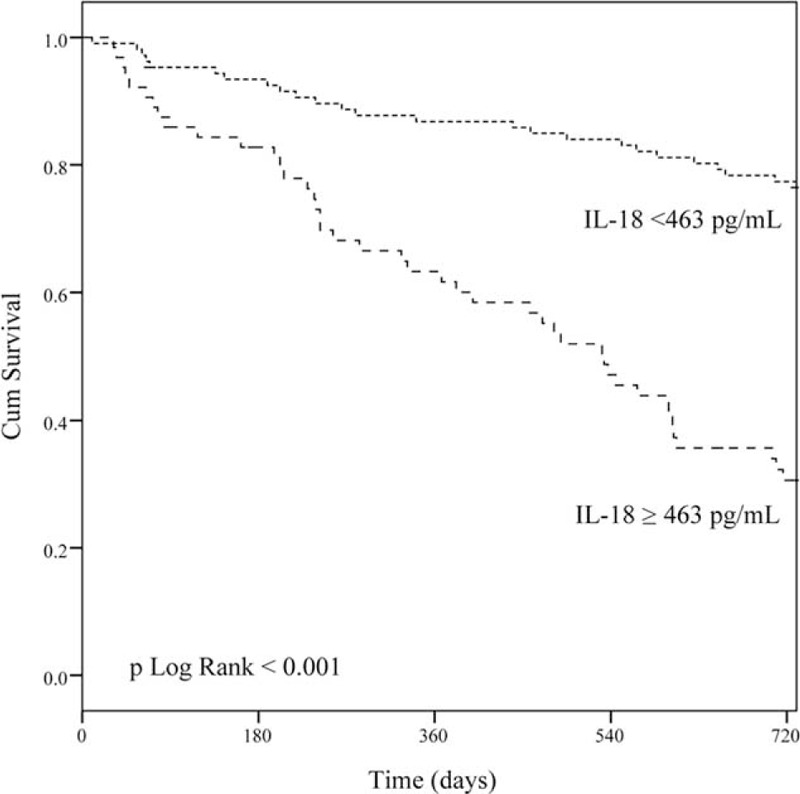
Cumulative event-free rate for 171 hemodialysis patients according to their IL-18 level.

Because the CRP level was associated with mortality in ESRD patients 6 months ago, the ability of the serum IL-18 level to predict patient outcome before disease onset was evaluated. In our study, the serum IL-18 level was more sensitive than the CRP level. Patients with an IL-18 level below 463 pg/mL exhibited markedly fewer cardiocerebral vascular events during the 2-year follow up (Fig. 2). Our finding suggests that IL-18 is promising biomarker to stratify the cardiocerebral vascular risk in hemodialysis patients.

Several limitations affected this study. First, this study was conducted in a referral center of a university in Northern Taiwan. Regional and ethnic differences must be considered. Second, only 1 measurement of the IL-18 level in this cross-sectional study was used to predict outcome. Previous studies have demonstrated a fluctuating release of inflammatory cytokines during the hemodialysis process. Therefore, repeat measurement after dialysis or a 3-month period may have improved the predictive ability. Third, gene polymorphism, binding proteins, and other cytokines that also belong to inflammatory cascades were not investigated; we examined only the correlation between the IL-18 level and cardiocerebral vascular events and the ability of the IL-18 level to predict these events. Fourth, the study population was small, and the current evidence was limited to include this parameter in cardiovascular risk prediction. Furthermore, validation study might be required. Finally, this study observed only a 2-year outcome; a long-term follow-up period is required to produce more conclusive findings. Combined examination of implicating pathways may improve the predictive power of a single IL-18 measurement. The assessment of IL-18 levels was expensive and time consuming. Before clinical application, additional prospective studies, including those of healthy populations, should be performed to standardize protocols and enable creating reproducible assays.

## CONCLUSIONS

In summary, IL-18 was found increased in dialysis patients who presented cardiovascular events, and could be considered as a possible predictor of cardiovascular events in this subgroup of patients. The results indicate the crucial role of inflammation as a negative prognostic factor in hemodialysis patients. It is noteworthy that various comorbidities might interfere the expression of IL-18; therefore, further validation study is required to incorporate IL-18 in clinical use.
